# Developing a Technique for the Imaging-Based Measurement of ACL Elongation: A Proof of Principle

**DOI:** 10.3390/diagnostics11112126

**Published:** 2021-11-16

**Authors:** Robert Csapo, Dieter Heinrich, Andrew D. Vigotsky, Christian Marx, Shantanu Sinha, Christian Fink

**Affiliations:** 1 Centre for Sport Science and University Sports, University of Vienna, 1150 Wien, Austria; 2 Department of Sport Science, University of Innsbruck, 6020 Innsbruck, Austria; dieter.heinrich@uibk.ac.at; 3 Departments of Biomedical Engineering and Statistics, Northwestern University, Evanston, IL 60208, USA; avigotsky@gmail.com; 4 Research Unit for Orthopaedic Sports Medicine and Injury Prevention, UMIT Tirol, 6060 Hall, Austria; christian.marx@umit.at (C.M.); c.fink@gelenkpunkt.com (C.F.); 5 Department of Radiology, University of California San Diego, San Diego, CA 92093, USA; shsinha@ucsd.edu; 6 Gelenkpunkt Sports and Joint Surgery, 6020 Innsbruck, Austria

**Keywords:** stress, strain, stiffness, mechanical properties, Lachman test, anterior tibial translation, MRI

## Abstract

Towards the goal of obtaining non-invasive biomarkers reflecting the anterior cruciate ligament’s (ACL) loading capacity, this project aimed to develop a magnetic resonance imaging (MRI)-based method facilitating the measurement of ACL elongations during the execution of knee stress tests. An MRI-compatible, computer-controlled, and pneumatically driven knee loading device was designed to perform Lachman-like tests and induce ACL strain. A human cadaveric leg was used for test purposes. During the execution of the stress tests, a triggered real-time cine MRI sequence with a temporal resolution of 10 Hz was acquired in a parasagittal plane to capture the resultant ACL elongations. To test the accuracy of these measurements, the results were compared to in situ data of ACL elongation that were acquired by measuring the length changes of a surgical wire directly sutured to the ACL’s anteromedial bundle. The MRI-based ACL elongations ranged between 0.7 and 1.7 mm and agreed very well with in situ data (root mean square errors, RMSEs ≤ 0.25 mm), although peak elongation rates were underestimated by the MRI (RMSEs 0.19–0.36 mm/s). The high accuracy of elongation measurements underlines the potential of the technique to yield an imaging-based biomarker of the ACL’s loading capacity.

## 1. Introduction

Tears of the anterior cruciate ligament (ACL) represent one of the most frequent orthopedic injuries in physically active cohorts. According to research investigating the mechanisms and risk factors for non-contact ACL injuries, various anatomical (e.g., decreased intercondylar notch width or increased tibial plateau slope), hormonal (pre-ovulatory phase in women), and neuromuscular (decreased hamstring, hip abductor, and knee external rotator strength) factors, as well as specific genetic predispositions (COL1A1 polymorphism or COL3A1 AA genotype), may increase a subject’s susceptibility to ACL tears [1,2]. With respect to the morphology of the ACL, ACL-injured subjects typically feature longer and narrower ligaments than healthy controls [3]. However, of even greater significance are the ACL’s mechanical properties, which are related to [4] but not solely determined by the ligament’s dimensions.

Unfortunately, obtaining reliable data reflecting the ACL’s stiffness is complicated. Clinicians often rely on measurements of knee joint laxity, such as the Lachman or anterior drawer tests [5], which may be assisted by the use of specific arthrometers [6]. However, both the accuracy and inter-rater reliability of these tests are poor [7,8]. Direct examinations of ACL stiffness were performed in vitro using cadaveric specimens [9] and in situ through the surgical implantation of strain transducers [10]. However, cadaver results cannot be readily extrapolated to in vivo conditions, and neither of these approaches is applicable in clinical routine. Some studies used medical imaging techniques, such as magnetic resonance (MR) or computed tomography, to obtain stress views of the knee joint during the application of anterior tibial translation loads [11,12]. However, considering that ligaments are viscoelastic tissues, images acquired under constant loading may not properly reflect their behavior under dynamic conditions. Novel imaging techniques to directly measure tissue stiffness, such as ultrasound shear wave or MR elastography, cannot be applied to the ACL due to its small size and/or anatomical location.

With the overarching goal to establish a measurement technique allowing for the assessment of ACL mechanical properties *in vivo*, this project aimed to (i) develop an MR imaging (MRI)-based technique to measure ACL elongations during the execution of Lachman-like knee stress tests and (ii) compare the imaging data to in situ measurements obtained from a human cadaveric specimen. Accurate elongation measurements may reflect the ACL’s loading capacity and represent the first step towards obtaining in vivo data of ACL stiffness.

## 2. Materials and Methods

### 2.1. Knee Loading Device

**Figure 1:**
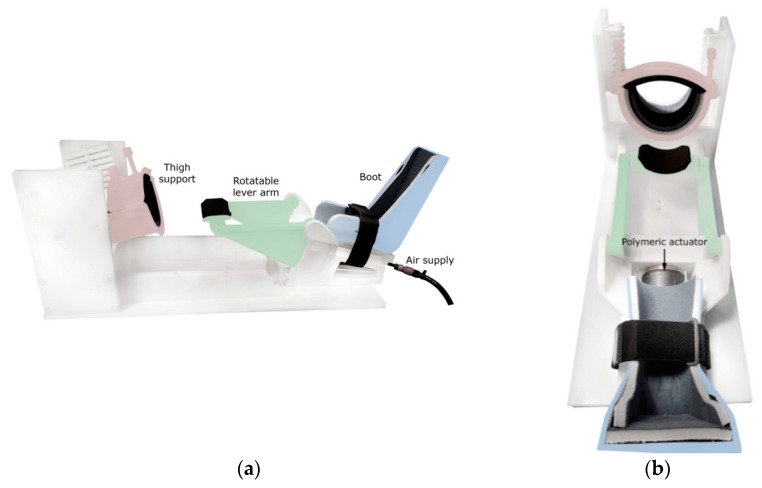
Pneumatic knee loading device as seen from the side (**a**) and top (**b**). Note the polymeric actuator beneath the boot.

To induce anterior tibial translation and perform Lachman-like knee stress tests within the MRI scanner, an MR-compatible knee loading device (see Figure 1) was designed and constructed by 3D-printing and subtractive manufacturing using rigid polyamide (PA12) and polyoxymethylene materials (Orthospect ACL; Ergospect GmbH, Innsbruck, Austria).

The device features support for the thigh and a semishell-shaped fixation that can be tightened using two bolts to properly immobilize the upper leg just proximal to the knee joint. The foot is placed in a designated boot, which is adjustable in the cranio-caudal direction for knee joint angle control. A Velcro strap may be wrapped around the ankle for additional fixation. Anteriorly directed forces may be pneumatically exerted on the posterior aspect of the lower leg using a system consisting of a polymeric actuator (Airomatic, Firestone Industrial Products LLC, Indianapolis, IN, USA) driving a rotatable lever arm. Upward rotation of the lever arm imposes anteriorly directed forces on the lower leg. Preliminary tests of the applicability of the device in vivo showed that subjects tolerate external forces of 200–250 N without experiencing undue discomfort in the area of force application just distal to the popliteal fossa (these force levels provoked maximal ACL elongations of ~2 mm; data not reported).

### 2.2. Experimental Setup and Stress Test Protocol

The core element of the measurement equipment is a control unit containing a Nucleo-64 board with a STM-32 microcontroller (Nucleo-G071RB, STMicroelectronics, Geneva, Switzerland). Positioned in the console room, it accepts user input from a laptop computer running custom-made software (Open JS Foundation, San Francisco, CA, USA). This tool serves to specify the forces applied during the stress tests, the rate and duration of loading, as well as the number of stress-relaxation cycles to be executed. Based on these parameters, the microcontroller regulates a pneumatic proportional valve (VPPC08, IMI plc, Birmingham, UK) that is connected to the knee loading device using a pneumatic tube looped through a penetration panel in the wall separating the console and scanning rooms. The air supply is provided by a wall-mounted pressurized air socket located in the scanning room.

**Figure 2:**
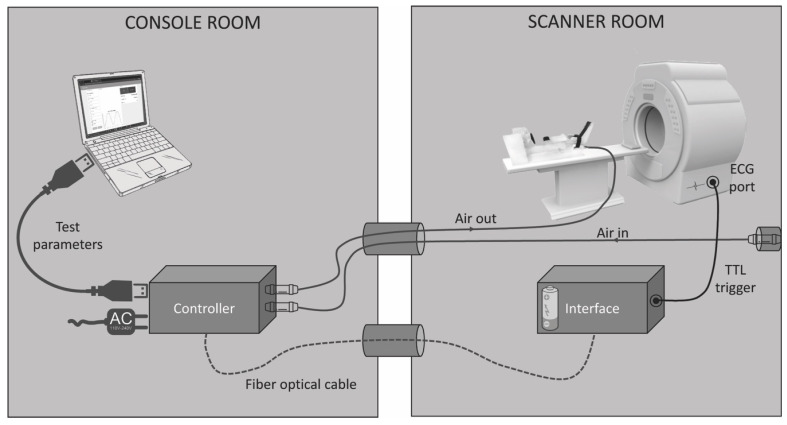
Experimental setup.

Apart from regulating the airflow to the knee loading device, the microcontroller also generates transistor-transistor logic (TTL) trigger impulses at times specified by the examiner using the software module. These trigger signals are then converted by a fiber optic converter (Meinberg Radio Clocks, Bad Pyrmont, Germany) and transmitted to a battery-powered interface located in the scanning room through a fiber optic cable. Here, the signals are converted back into TTL impulses (Meinberg Radio Clocks) and fed into the scanner’s electrocardiogram port to trigger image acquisition. The experimental setup is shown schematically in Figure 2.

For our cadaveric experiment, the right leg of a 56-year-old, male Caucasian was used. The specimen was stored at −20 °C until 24 h before testing and then slowly thawed to room temperature. To provoke ACL elongations, the following protocol was used: First, the externally applied forces were increased over 5 s from a baseline of 20 N to maximal forces of 300, 350, and 400 N, respectively. The slightly elevated baseline force level was deliberately chosen as the motion of the knee loading device was much smoother when the system was constantly kept under pressure. It must also be pointed out that the maximal forces used in the cadaver test were higher than those we typically apply in in vivo tests (200–250 N; see previous section). This is because we found the cadaveric leg to be stiffer, necessitating the application of higher maximal stimulation forces to provoke maximal ACL elongations of similar dimensions (~2 mm; see previous section). Following the period of force increase, forces were maintained at the maximal level for a further 5 s (plateau region) before being reduced back to baseline at the same rate (5 s). Individual stress-relaxation cycles were separated by a rest period of 2 s.

### 2.3. MRI

For MRI, subjects are usually placed on the examination bed in the supine and feet-first position with the examined leg firmly fixed in the knee loading device at a 20° knee angle. In our cadaveric experiment, the semishell-shaped fixation for the thigh could not be used for appropriate fixation. Therefore, the residual thigh was fixed by two bolts drilled through the femur in the anterior-posterior and latero-medial direction and directly screwed onto the knee loading device. The rotatable lever arm of the device was positioned just distal to the popliteal fossa. A 3T whole-body scanner (Skyra, Siemens Healthineers, Erlangen, Germany) was used. Following the acquisition of two sets of localizers in the coronal and axial plane, one stack of high-resolution images was acquired in a parasagittal plane using a T1-weighted TSE sequence (15 slices, thickness 2.5 mm, spacing 2.5 mm, TE 10 ms, TR 700 ms, NEX 1, ETL 3, FOV 192 mm × 220 mm, and 336 × 384 matrix). This served to identify the single slice providing the best visualization of the ACL in its longitudinal direction. Using the orientation and position of this slice, a real-time cine GR sequence was acquired using the following scanning parameters: thickness 8 mm, TE 1.5 ms, TR 104 ms, FOV 192 mm × 220 mm, 126 × 144 matrix, 96 phase encoding steps, NEX 1, and ETL 1. With the larger slice thickness, both the femoral and tibial footprints of the ACL remained clearly distinguishable despite potential mediolateral shifts occurring during test execution. This sequence yielded a total of 511 images acquired throughout 53 s (9.6 Hz), allowing for three consecutive stress-relaxation cycles to be fully captured.

### 2.4. MRI Post-Processing

**Figure 3:**
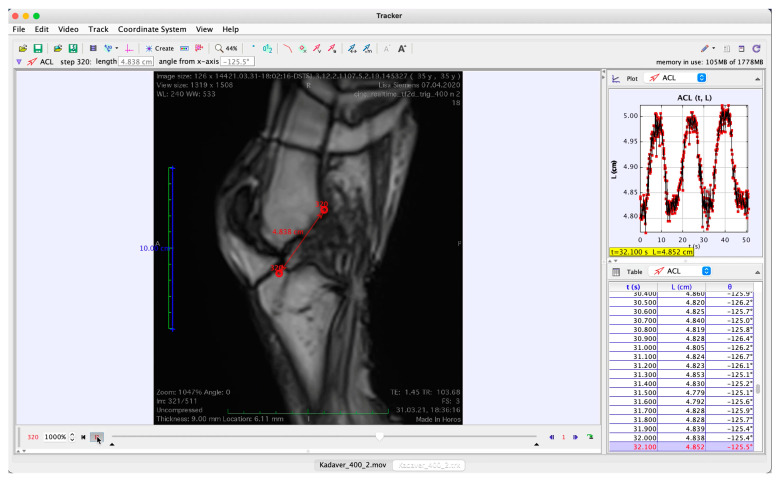
Screenshot of MRI cine sequence with points tracked for the estimation of ACL elongation. The red dots represent the points tracked during test execution.

The MR images were converted into a movie sequence (Horos 4.0, Nimble Co., LLC, Annapolis, MD, USA; http://www.horosproject.org, accessed on 16 November 2021) for import into a tracking software (Physlets Tracker 5.1.5, Open Source Physics, https://physlets.org/tracker, accessed on 16 November 2021), which was used to automatically track two points located at distinctive bony structures near the ligament’s femoral and tibial footprints; erroneous trackings were manually corrected. A demonstrative screenshot of an image from the MR cine sequence with the tracked points superimposed is shown in Figure 3 and the full video is provided as Supplementary Video S1.

**Figure 4:**
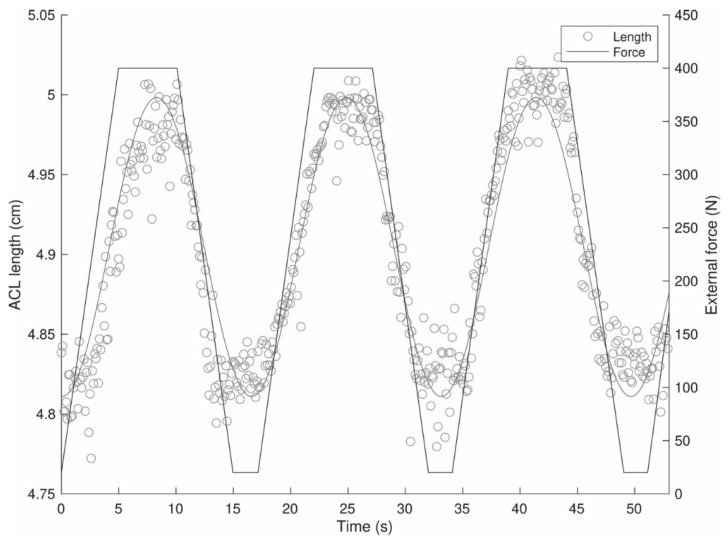
Demonstrative time-length and time-force data series.

The coordinates of the ACL endpoints were then imported into MATLAB (R2019b, The Mathworks Inc., Natick, MA, USA) and used to calculate the ACL length as the Euclidian distance between the points. The length measures were then fitted using a smoothing spline (smoothing parameter *p* = 0.5). Figure 4 depicts a demonstrative length-time and corresponding force-time data series from the cadaveric test. Finally, ACL elongations were calculated by subtracting the minimal ACL length (considered to represent the resting length) measured during the respective trial.

### 2.5. In Situ Measurements of ACL Elongation

**Figure 5:**
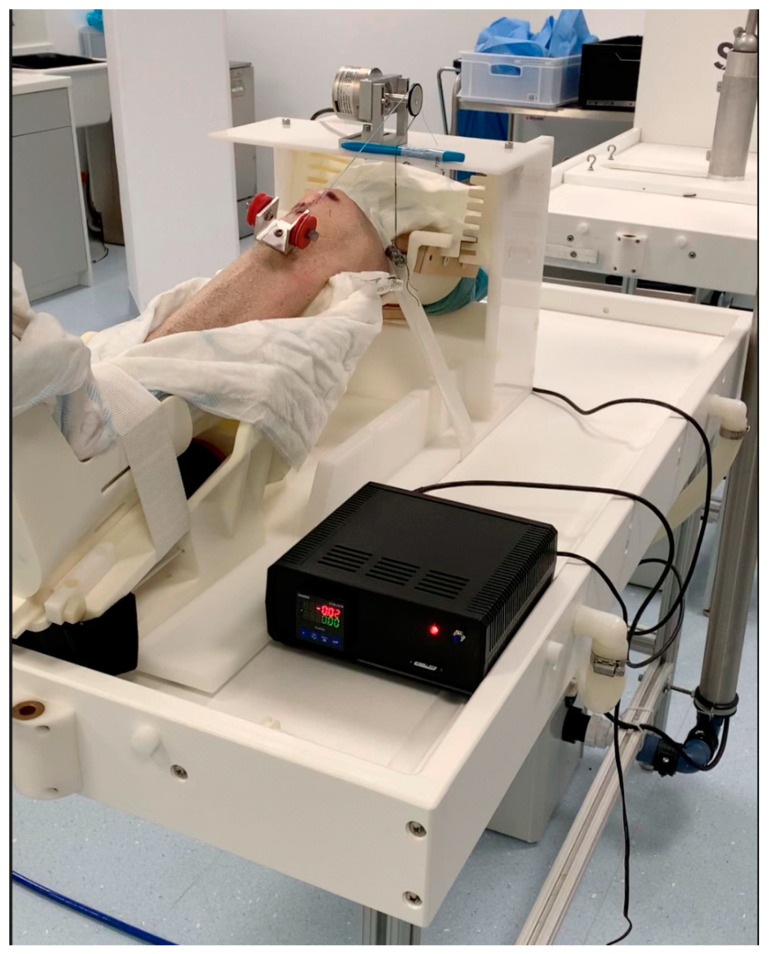
Measurement setup for cadaver tests.

The MRI-based elongation data were compared to in situ measurements obtained in the same cadaveric model. A 5.5 mm Bio-Corkscrew^®^ (Arthrex GmbH, Munich, Germany) was arthroscopically placed within the femoral footprint of the ACL (anteromedial aspect) through a standard anteromedial portal. One No. 2 FiberWire^®^ suture (Arthrex) was removed and the other was retrieved through the medial portal. Using a standard ACL tibial aimer, a 2.5 mm k-wire was placed in the tibial footprint of the ACL (anteromedial aspect). The k-wire was removed and a suture passing wire (Arthrex) was inserted. The wire was also retrieved through the anteromedial portal. The FiberWire suture was then shuttled through the tibial tunnel. A pulley system was mounted on the tibia, aligned with the direction of the tunnel, and fixed to the tibial shaft using a 4.5 mm cortical screw. The suture led through the tibial drill hole was then passed around the pulley and placed on top of the wheel of a rotary potentiometer (PRID 58H8, Opkon Ltd., Istanbul, Turkey), which was mounted on the knee loading device over and slightly proximal to the knee joint. Lead weights were attached to the end of the suture to warrant sufficient friction between the wire and the potentiometer, the signals of which were digitized through an interface (EZM-4430, Emko Elektronik Gmbh, Planegg, Germany) and saved onto a memory card. Thus, the extent of suture extension induced through the execution of computerized Lachman tests using the knee loading device could be registered with an accuracy of 0.01 mm. Figure 5 shows the cadaver test setup. Following the completion of in situ measurements, all ferromagnetic components were removed before repeating the ACL elongation measurements in the MRI scanner as detailed above.

### 2.6. Statistical Analyses

For the comparison of ACL elongation data acquired in situ and through MRI, the data corresponding with the rising sections in force curves were extracted, modeled using a logistic function and fitted using generalized nonlinear least squares with AR(1) errors to account for temporal autocorrelation and appropriately adjust parameter variance estimates [13]. Logistic functions fit the data well (see Section 3) and have interpretable parameters that represent salient features of the elongation curves; namely, peak elongation (*A*) and peak elongation rate (*k*). The model also captures the time of 50% strain, but this was not of interest and thus treated as a nuisance parameter. Each elongation curve was modeled separately since we were interested in absolute agreement rather than average differences.

(1) $$\left(t\right)=\frac{A}{1-exp\left\{\frac{-4k}{A}\left(t-t_{0.5}\right)\right\}},$$

After obtaining the parameter estimates and their variances, the in situ elongation means and their corresponding standard deviations (SD) were estimated using inverse variance weighting—the weighted mean served as the gold standard. MRI-based estimates were compared to the in situ measurements using inverse-variance weighted root mean squared errors (RMSEs). Confidence (or compatibility) intervals (CIs) of 95% were calculated using a stratified bias-corrected and accelerated bootstrap with 1000 replicates, for which both in situ and MRI data were simultaneously resampled with replacement to account for both sources of variance.

## 3. Results

**Figure 6:**
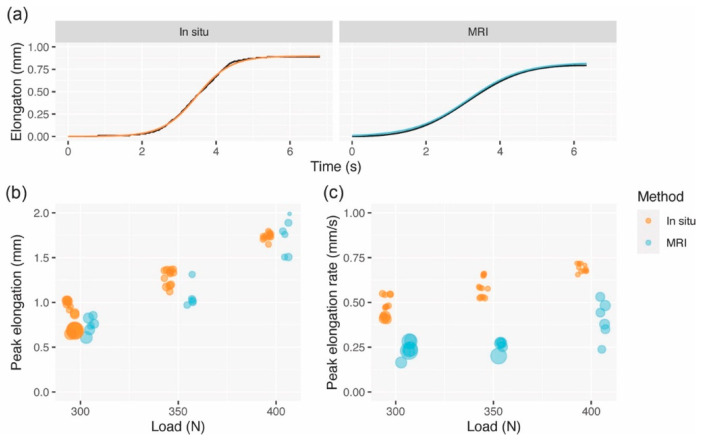
Results from logistic models. (**a**) Representative curves from a single rise cycle using 350 N; (**b**) Peak elongations from the in situ and MRI methods; and (**c**) Peak elongation rates from the in situ and MRI methods. Dot sizes (**b**,**c**) are in scale with inverse variance weighting.

The maximal elongations as measured in situ ranged between 0.7 (300 N) and 1.7 mm (400 N). Logistic models fit the data well (all R^2^ > 0.99), enabling us to compare elongations and elongation rates between the data obtained in situ and through MRI. There was generally good agreement between peak elongations from the in situ and MRI-based measurements, with RMSEs that were an order of magnitude smaller than the deformations themselves. In contrast, peak elongation rates demonstrated poor agreement, with elongation rates measured by MRI being markedly lower and RMSEs on the same order as the peak elongation rates themselves. The elongations and elongation rates as well as the respective MRI RMSEs are summarized in Table 1 and the results are depicted graphically in Figure 6.

## 4. Discussion

With the long-term goal of obtaining in vivo estimates of ACL mechanical properties, the present study aimed to develop an MRI-based technique for the assessment of ACL elongation during the execution of Lachman-like stress tests. Using a custom-made knee loading stress test device and a triggered MRI cine sequence, we measured ACL elongations in a cadaveric specimen for comparison with in situ measurements obtained through a surgical wire directly sutured to the ACL. The results of our study showed that the peak ACL elongations measured with the MRI-based protocol agreed well with in situ data. Peak elongation rates, by contrast, were markedly lower, indicating the lower responsiveness of MRI measurements.

Several earlier studies have attempted to measure ACL strains and/or mechanical properties in vivo. Taylor et al. [14] developed an elegant approach that relied on the application of MRI to create three-dimensional bone models of the knee, which they then used in combination with motion capture and biplanar fluoroscopy to assess ACL strains during walking. More recently, the use of high-speed radiographs has further improved the accuracy of ACL deformation measurements [15]. While these experiments investigated the ACL’s dynamic function in either everyday tasks or athletic activities, other studies were specifically designed to determine ACL stiffness. For example, Kang and colleagues [11] used computed tomography images obtained during the execution of drawer tests to measure tibial translation and generate non-linear finite element models of the knee joint. Ligament stiffnesses were then mathematically optimized to minimize the differences between the tibial translations in the finite element models and medical images. Another modeling-based approach was adopted by Westover et al. [12] who obtained the stress views of the knee by MRI, thus avoiding radiation exposure. However, to the best of our knowledge, our study is the first attempt to develop a measurement technique that allows for the real-time visualization of ACL elongation in response to increasing loads.

In developing the measurement technique, several challenges had to be overcome. To warrant MR-compatibility, the knee loading device had to be constructed exclusively of non-ferromagnetic, yet sufficiently rigid materials. For this purpose, a combination of polyamide and polyoxymethylene materials was processed using 3D-printing and subtractive manufacturing techniques. Moreover, power cables in the vicinity of the MR scanner may induce magnetic field inhomogeneities. To minimize the risk of associated imaging artifacts, the knee loading device was driven pneumatically and synced with the MRI scanner through a fiber optic cable and a galvanically isolated interface located in the scanning room. The dynamic MRI sequence had to satisfy both the needs for adequate temporal and spatial resolution and ensure the visualization of the ACL in its entire length despite potential mediolateral shifts during stress tests. These demands were met by using a real-time cine GR sequence with a temporal resolution of approximately 10 Hz and a slice thickness of 8 mm. The tracking of ACL endpoints in the resulting cines was complicated by the fact that the ACL appears as signal-void in routine MRI. For this reason, we decided to track distinct bony structures near the ACL footprints instead.

The resultant elongation measurements were reasonably reproducible, sufficiently sensitive to capture the small ACL length changes (0.70–1.69 mm), and—as evidenced by RMSEs ranging between only 0.09 and 0.25 mm—agreed very well with in situ data. The reasons for the discrepancies of length change velocities (MRI consistently underestimated maximal elongation rates) are unclear. One possible explanation is that the surgical wire used for in situ tests was attached to the ACL’s anteromedial bundle, which acts as the primary restraint to anterior tibial translation [16]. With an 8 mm slice thickness, the MR images, by contrast, probed the ligament’s entire cross-sectional area. Possibly, the simultaneous scanning of more and less strained components introduced bias, giving the impression of an overall slower elongation. In addition, the MRI itself may act as a low-pass temporal and spatial filter since its resolution is poorer than that of in situ measurements. Another potential source of error lies in the algorithm of the tracking program used. The software allows the user to specify the evolution rate, which quantifies the degree to which the templates to be tracked may change in shape and color over time. Setting this parameter overly conservatively (i.e., to too low values) impedes the tracking since template changes are inherent in dynamic MRI. Higher evolution rates, however, may result in minor template drifts. While care was taken to set the evolution rates to the minimal values permitting persistent tracking and all tracks were visually inspected, it cannot be ruled out that this procedure introduced some delay in elongation measurements. However, assuming acceptable reliability, the technique presented in this paper may still be used to obtain estimates of ACL elongation, even if the agreement of strain rates is less than optimal. Follow-up in vivo studies will establish this arguably most important test criterion.

In the future, we hope to not only be able to measure ACL elongation but also to estimate the ligament’s stiffness. For this purpose, in addition to the elongation measurements, the forces acting on the ACL must be accurately determined. In in vivo studies, ACL forces may only be estimated through the use of biomechanical models. Examples of rather complex models that take the subject-specific anatomy of the knee joint into account have been given above. One example of a simpler, generic knee model that might easily be applied in clinical routine is that proposed by Nasseri et al. [17]. The model is based on cadaveric data [18,19] and may be used to estimate ACL forces as a function of uniplanar (anterior drawer) force and knee joint angle. However, considering that in vitro biomechanical testing to validate estimates of ACL stiffness was not available in this study, we decided not to report the respective data.

This study is subject to several limitations. First, it must be pointed out that the fixation of the cadaveric leg differed from that intended for living subjects. This is because the residual thigh could not be properly immobilized using the semishell-shaped fixation of the knee loading device and, thus, had to be fixed using two additional bolts directly drilled through the femur. Further, the MRI sequence used for elongation measurements displays the ACL as a signal-void structure. This is because the rigid binding of hydrogen atoms in collagenous tissues results in very short transversal relaxation times and fast signal decay. Such tissues are presented as signal-void structures and inherently difficult to track unless one uses Ultra-short TE sequences which can render fibrocartilaginous structures bright but which unfortunately are currently too long to allow cine dynamic imaging. Hence, we decided to track distinctive bony structures near the ACL’s footprints on the femur and tibia instead. In the future, the development of dynamic MRI sequences with ultra-short echo times may help circumvent this limitation [20]. As mentioned above, the reasons for the discrepancies in maximal elongation rates between in situ and MRI-based data remain unclear. It seems plausible that the characteristics of our image acquisition protocol and the algorithm of the tracking software could have introduced bias resulting in a slower rise of MRI-based force-elongation curves, but this warrants further investigation. Finally, although the analysis of several loading cycles obtained at different levels of external force indicated acceptable reproducibility of the MRI-based ACL elongation measures, more thorough reliability analyses in larger samples and under in vivo conditions have yet to be conducted.

## 5. Conclusions

In conclusion, we here propose a measurement technique to perform Lachman-like knee stress tests in MR scanners and measure the resultant ACL elongations. The measurement system consists of a pneumatically driven, computer-controlled, and MR-compatible knee stress test device that generates trigger impulses to synchronize the acquisition of gated cine-images with stress-relaxation cycles. A real-time cine GR sequence that yielded a temporal resolution of ~100 ms per image was used for image acquisition. The imaging-based ACL elongation measures acquired in a cadaveric model were compared to in situ measurements obtained by monitoring the length changes of a surgical wire directly sutured to the ACL’s anteromedial bundle. The comparison of results confirmed the good agreement of maximal elongations, although maximal elongation rates were underestimated by MRI. Following further reliability tests, this technique will be applied in vivo to track changes in the ACL’s loading capacity over time.

## Figures and Tables

**Table 1:** Comparison of in situ and MRI-based measurements of maximal ACL elongations and elongation rates.

Peak External Force	Maximal Elongation (mm)			Maximal Elongation Rate (mm/s)		
	In Situ Data	MRI Data	MRI RMSE (95% CI)	In Situ Data	MRI Data	MRI RMSE (95% CI)
300 N	0.70 ± 0.07	0.70 ± 0.09	0.09 (0.05, 0.13)	0.43 ± 0.04	0.24 ± 0.03	0.19 (0.16, 0.23)
350 N	1.27 ± 0.09	1.04 ± 0.09	0.25 (0.18, 0.30)	0.58 ± 0.05	0.22 ± 0.03	0.36 (0.30, 0.40)
400 N	1.73 ± 0.03	1.69 ± 0.17	0.17 (0.10, 0.23)	0.69 ± 0.02	0.42 ± 0.08	0.28 (0.22, 0.35)

In situ and MRI values are presented as the weighted mean ± weighted standard deviation from the logistic model fits. The MRI root mean square errors (RMSEs) and their 95% confidence intervals (CIs) are similarly weighted.

## Data Availability

The data presented in this study are openly available on the Open Science Framework (https://osf.io/fahcg/, accessed on 16 November 2021).

## Supplementary Materials

The following are available online at https://www.mdpi.com/article/10.3390/diagnostics11112126/s1: Video S1: Demonstrative video showing the magnetic resonance cine sequence and the tracking of points close to the anterior cruciate ligament’s femoral and tibial footprints.
